# Effects of Hormone Therapy on Oxidative Stress in Postmenopausal Women with Metabolic Syndrome

**DOI:** 10.3390/ijms17091388

**Published:** 2016-08-24

**Authors:** Martha A. Sánchez-Rodríguez, Mariano Zacarías-Flores, Lizett Castrejón-Delgado, Ana Karen Ruiz-Rodríguez, Víctor Manuel Mendoza-Núñez

**Affiliations:** 1 Unidad de Investigación en Gerontología, Facultad de Estudios Superiores Zaragoza, Universidad Nacional Autónoma de México, Guelatao No. 66, Col. Ejército de Oriente, Ciudad de México, México CP 09230, Mexico; masanrod@yahoo.com.mx (M.A.S.-R.); lizettcastrejon@hotmail.com (L.C.-D.); hikary90@hotmail.com (A.K.R.-R.); 2 División de Ginecología y Obstetricia, Hospital General Dr. Gustavo Baz Prada, Instituto de Salud del Estado de México, Av. Bordo de Xochiaca esq. Adolfo López Mateos S/N, Col. Tamaulipas, Nezahualcóyotl, Estado de México, México CP 57300, Mexico; mzacariasf@yahoo.com

**Keywords:** oxidative stress, hormone therapy, postmenopause, metabolic syndrome, lipids profile

## Abstract

The aim of this study was to determine the effect of oral hormone therapy (HT) on oxidative stress (OS) in postmenopausal women with metabolic syndrome (MetS). A randomized, double blind, placebo-controlled trial was carried out. We formed four groups of 25 women each; healthy (HW) and MetS women (MSW) were assigned to HT (1 mg/day of estradiol valerate plus 5 mg/10 day of medroxiprogesterone) or placebo. We measured plasma lipoperoxides, erythrocyte superoxide dismutase and glutathione peroxidase, total plasma antioxidant status and uric acid, as OS markers. Alternative cut-off values of each parameter were defined and a stress score (SS) ranging from 0 to 7 was used as total OS. MetS was defined according to National Cholesterol Education Program Adult Treatment Panel III (NCEP-ATPIII) criteria. Participants were seen at baseline, 3 and 6 months. After 6 months, MetS decreased in MSW-HT (48%), their triglycerides and high-density lipoprotein cholesterol (HDL-c) improved; in the other groups no difference was found. SS in MSW-HT decreased (3.8 ± 0.3 to 1.7 ± 0.3, *p* < 0.05) and OS was also reduced (44%), this effect was evident since 3 mo. HW-HT with high OS also decreased (40%). In placebo groups there was no change. Our findings suggest that HT improve lipids and OS associated to MetS in postmenopausal women.

## 1. Introduction

It is known that metabolic syndrome (MetS) is a set of physical, biochemical and hematological alterations that included abdominal obesity, insulin resistance, dyslipidemia with high triglycerides (TG) and low high-density lipoprotein cholesterol (HDL-c) concentration, high glucose concentration, blood hypertension and a prothrombotic state, which encourage an increased risk of cardiovascular morbidity and diabetes [1]. Several organizations have proposed different definitions, but the National Cholesterol Education Program definition, called NCPE Adult Treatment Panel III (NCEP-ATP III) is the one more widely used [2].

The characteristic metabolic changes of MetS worsen due to the endocrine changes that occur during aging, particularly in women after menopause [3]. In this regard, it has been reported that as a result of the decrease in estrogen level, an increase in abdominal adiposity and a decrease in energy expenditure occurs; this causes an increase in the cholesterol and TG concentration, in addition to a significant increase in glucose and insulin concentrations, which may explain the increased frequency of MetS and the risk of cardiovascular disease and death during the post-menopausal period [4,5].

Likewise, it is known that MetS is related to aggravated oxidative stress (OS), both in animal models and in postmenopausal women [5,6,7], mainly due to hyperinsulinemia and the lack of control of glycaemia. Links between atherosclerosis along with cardiovascular risk and MetS, insulin signaling, OS and inflammation have been reported [6,7,8].

OS is the biochemical imbalance caused by the excessive production of reactive species, mainly of oxygen, that cannot be counteracted by antioxidant systems which causes oxidation of biomolecules [9] and has been implicated in more than one hundred chronic diseases. In this context, previously we reported that MetS is associated with severe OS and the number of components of MetS is a risk factor for oxidative imbalance during aging [10]; also, we have shown that menopause is a risk factor for OS and that the severity of postmenopausal symptoms increases OS in postmenopausal women with MetS [11,12].

Estrogen exerts actions both as an antioxidant molecule and as a sexual hormone due its structure and its capacity to prevent OS in different ways, thus estrogen mitigates OS [13,14]; so, during the postmenopausal period when estrogen levels fall, this antioxidant’s protection is lost. Hormone therapy (HT) with estrogen and progestin or with estrogen alone is a therapeutic alternative for women seeking help to control their symptoms that occur after menopause, although the therapy use is controversial, it is actually accepted if its use is near menopause [15]. In this sense, HT has shown positive effects in women with MetS by producing a significant reduction in insulin resistance, hyperinsulinemia and visceral fat and better management of weight, hypertension and diabetes mellitus [4,16]. Regarding OS, several studies have been conducted to determine the antioxidant effect after menopause, although there are no reports about this effect on OS in women with MetS; hence, the purpose of this study was to determine the effect of HT on OS in postmenopausal women with MetS.

## 2. Results

### 2.1. Participants Disposition and Characteristics of Study Groups

**Figure 1:**
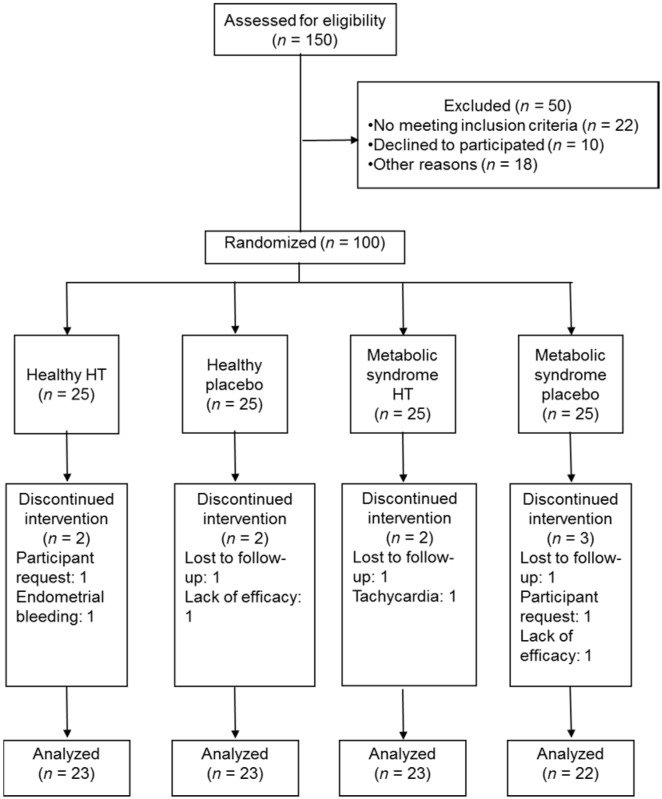
Diagram of follow-up of the women during the clinical trial. HT: hormone therapy.

Among the participants in the study, nine women from the four groups did not complete the follow-up. The rates of discontinuation were 10% in the placebo and 8% in the HT groups. In the placebo groups the women reported lack of efficacy, therefore they dropped out. In the HT groups two women reported clinical discomfort and other participants discontinued treatment for personal reasons. Finally, 22 women in the placebo MSW group and 23 in each of the other groups completed the study (Figure 1).

Baseline parameters were similar among the study groups with the exception of the body mass index between placebo and HT groups in healthy women (*p* < 0.05). There were no statistically significant differences in OS markers at baseline among the four groups (Table 1).

Using the cutoff of metabolic alterations according to NCEP-ATP III criteria, we observed that the prevalence of these characteristics were similar among treatment groups. Also, no differences were found among groups in pro-oxidant factors (Table 2).

### 2.2. Effects of Hormone Therapy on Metabolic Syndrome Markers

After 6 months of treatment, only 11 (48%) MetS women (MSW) taking HT continued with the disease (*p* < 0.05), while 20 (91%) women in the placebo group remained with this. Likewise, 2 (9%) of the healthy women (HW) group taking HT and 5 (22%) of the placebo group developed MetS.

HT significantly reduced the TG concentration (2.42 ± 0.18 vs. 2.02 ± 0.17 mmol/L, *p* < 0.05) and HDL-c concentration showed a no significant increase (1.33 ± 0.08 vs. 1.43 ± 0.07 mmol/L) from baseline in MSW after 6 months; there were no differences on the rest of MetS markers. The other three groups did not show change in all the MetS parameters measured. Also after 6 months, HT reduced low density lipoprotein cholesterol (LDL-c) concentration in the two groups with treatment (MSW 3.5 ± 0.26 vs. 2.6 ± 0.24 mmol/L, *p* < 0.001; HW 3.0 ± 0.16 vs. 2.5 ± 0.13 mmol/L, *p* = 0.001).

The proportion of women with hypertriglyceridemia decreased in MSW taking HT from 19 participants (83%) to 13 (57%), a 26% change (*p* < 0.05); and the proportion of women with hypoalphalipoproteinemia also diminished from 13 participants (57%) to 9 (39%)—an 18% change—but it was not statistically significant. In HW with HT, we only observed a decrease in the proportion of women with hypertension, from 7 participants (30%) to 3 (13%), a 17% of change (*p* > 0.05). In placebo groups, the percentage change was positive, or without changes on the MetS markers (Table 3).

### 2.3. Effects of Hormone Therapy on Oxidative Stress Related With Metabolic Syndrome

Lipid peroxides (LPO) concentration significantly diminished in both groups with HT after 6 months. Glutathione peroxidase (GPx) and antioxidant gap, as antioxidant components increased and superoxide dismutase/GPx (SOD/GPx) ratio was reduced in MSW with HT. In HW with HT, only GPx was significantly higher when compared with the baseline activity. In the placebo groups, there was no change across the timespan (Table 4).

**Figure 2:**
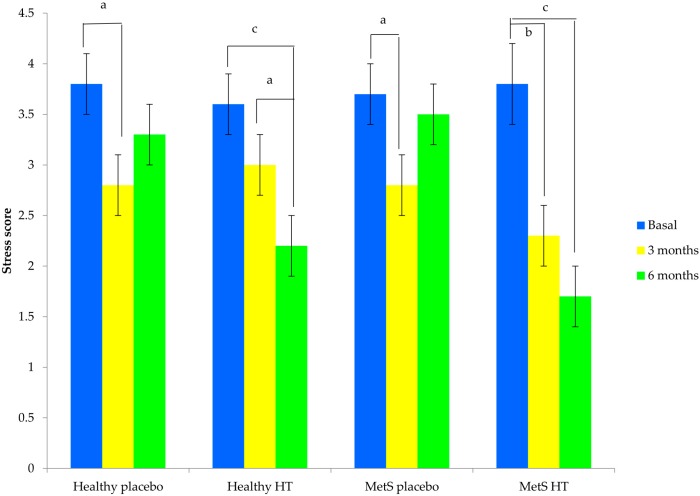
Stress score in the three moments of measure by the study group. Data is expressed as a mean ± standard error. Repeated measure analysis of variance (ANOVA) test, time by group (*p* < 0.01). Paired *t*-test: ^a^ *p* < 0.05; ^b^ *p* = 0.001; ^c^ *p* < 0.0001. MetS: metabolic syndrome; HT: hormone therapy.

Oxidative stress score (SS) declined during the course of the study in the two groups with HT, but the effect was highest in the women with MetS because a significant reduction of SS was observed as early as 3 months; in HW, the antioxidant effect was seen until month 6. In both placebo groups, SS decreased at month 3 but increased again at end of follow-up (Figure 2).

**Figure 3:**
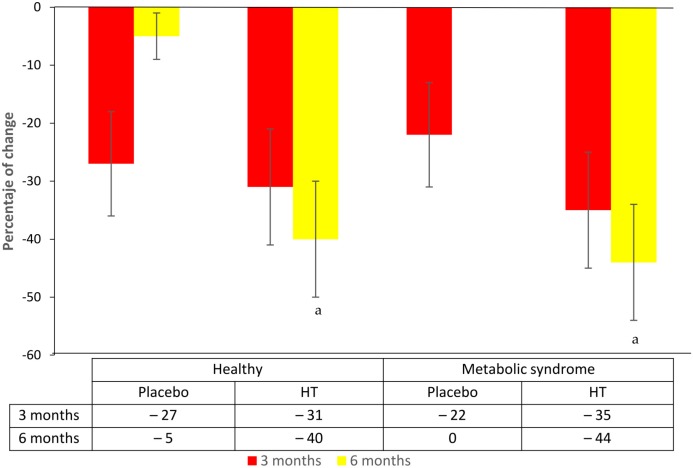
Percentage change of oxidative stress (stress score ≥4) after 3 and 6 months of treatment. Error bars are proportion standard error. McNemar *chi* *square* test. ^a^ basal vs. 6 mo., *p* < 0.05; HT: hormone therapy.

Also, the percentage of women with OS changed according to SS cut-off and was negative in both groups of women with HT; but it was more favorable in MSW when compared to healthy ones (−44% vs.−40%). In the placebo groups, the OS diminished at month 3, returning to almost the basal proportion in both (Figure 3).

## 3. Discussion

Metabolic changes during the menopausal transition and the increase in abdominal adiposity due to reduced estrogen production resulting in the absolute or relative increase in free androgens, may explain the incidence of MetS during this period [3]. It has been reported that the prevalence of MetS in postmenopausal women oscillates between 30% to 40% according to NCEP-ATP III criteria [17,18,19], which is quite significant because in the past decade this figure has increased and it is estimated that women with MetS had a high risk to cardiovascular diseases [20].

In this study we observed that increased waist circumference is a common finding in the participants and healthy women have a high risk of developing MetS due to the abdominal adiposity, as noted above. In this respect, it has been reported that the risk of developing MetS is 2.75 times higher in overweight individuals and 7.8 times higher in obese individuals [19]. In women, this increase in abdominal adiposity is explained by the establishment of an androgenic environment due to hypoestrogenism; it has been reported that for each increase of one standard deviation in the circulating level of bioavailable testosterone, there is a 10% increase in the risk for development of MetS [21].

It is proposed that the MetS is a state of chronic low-level inflammation, regardless of body weight, which is associated with OS [8,22]; however, our study groups are very similar in all the parameters, with a no significant increase of LPO concentration. A possible explanation of these contradictory results is a high prevalence of obesity in the groups because it has been reported that fat accumulation is related with OS [23] and the decrease in estrogen level after menopause leads to changes in the lipid profile and as a consequence, to an increase of lipid peroxidation [24,25]. Considering that OS is our dependent variable, we used an oxidative stress score (SS) that integrates both oxidant and antioxidant markers to represent the dynamics of OS. This index included LPO levels and SOD/GPx ratio as oxidative damage markers, two antioxidant enzymes (superoxide dismutase (SOD) and GPx) and three plasma antioxidant, as a way to completely evaluate the OS, so that we may observe the HT effect over OS.

The aim of this study was to determine the effects of HT on MetS and the OS-related. Then, a decrease in the incidence of MetS was observed in women treated with HT, of whom 52% still experienced MetS after six months of treatment, thus we saw 48% of treatment effectivity. According to our results, HT affects the components of the lipid profile, mainly triglyceride and HDL-c levels, in which the percent of change was higher. The results of the effects of HT with estrogen on postmenopausal women has been noted; then it is reported, a positive effect on HDL-c in healthy and MetS women, as we have previously mentioned [4,16,26,27]. Moreover, a study indicated that with HT there is an increase in Apo A–I production, which was not accompanied by a corresponding increase in HDL-c [28].

Regarding TG, the concentration clearly decreased in the group of women with MetS taking HT, while in the other groups, no change or an increase over time were observed. Several studies have demonstrated that when administering HT with estrogen, a decrease in TG concentration is observed, although without a statistically significant difference or with a neutral effect, this may be due to their use, alone or combined with different progestin; likewise, these studies do not highlight the presence of MetS in their participants [26,28,29]. These authors argue that although endogenous estrogen exerts greater activity on lipids than exogenous estrogen, they do neglect the idea that the latter may retain some activity on the lipid profile. This is contrary to the idea proposed at the beginning of the investigation regarding the effects of HT on the lipid profile, which indicated that treatment caused an increase in triglyceride levels [30]. Actually, we have known that hypertriglyceridemia enhances the cholesteryl ester transfer protein (CETP)-mediated interchange of TG from TG-rich lipoproteins to HDL particles and the subsequent TG-enrichment of the HDL particle. Hepatic lipase has greater activity against TG and will therefore convert large HDL particles to small HDL particles, which are cleared more rapidly from the circulation by the kidney, thus reducing the HDL-c concentration. Therefore, reductions in TG levels as we saw with HT, will increase HDL-c concentration [31]. Given these findings, our results support the notion that there is a beneficial effect of HT as a complementary treatment for MetS in postmenopausal women, emphasizing the benefit on the lipid profile more than on other markers because it can reduce the risk of cardiovascular events.

On the other hand, sufficient evidence has emerged that shows that the relationship between MetS and OS is an associated abnormality [10,32]. We have previously reported that women with MetS have a higher level of OS compared to healthy and it is even higher if these women have severe menopausal symptoms [12]. In this study we did not see this phenomenon and this can be partially explained due to the small sample size.

Moreover it is known that estrogen, with or without progestin, is effective in treating menopausal symptoms, in addition to its antioxidant effect demonstrated in vitro and in vivo studies. This is probably due to its chemical structure which inhibits or neutralizes excess of reactive oxygen species by different mechanisms [13,14]. This antioxidant effect is not reported in MSW therefore, our interest is to also determine if OS decreases in postmenopausal women with MetS using HT in order to provide an additional benefit to this therapy.

In this study, we found that the difference in the SS is evident in the third month of follow-up, with a decrease in all groups, but this effect was more pronounced in women with MetS and HT. After six months, the effect on this last group was more evident and the proportion of MSW with OS diminished at 44%. That is, there is an improving of OS greater than the one seen in healthy women undergoing HT and contrary to the null effect in the groups that received the placebo. Considering these results, we observed the same outcome of our previous report that shows a beneficial effect of HT on OS in post-menopausal women [33] confirming its antioxidant action; although this effect is most evident in those with high levels of OS such as women with MetS. Regarding this idea, some authors suggest that the first and most noticeable difference related to anti-OS therapy occurs in the level of lipid peroxides because lipid peroxidation is a process induced by ROS and has been widely studied [13,34]. A difference found in the current study compared with previous research was the effect observed on a stress score index, composed of oxidant and antioxidant markers and not of individual markers, which represents the dynamic nature of the process. This result is feasible because estrogen modulates both oxidative and antioxidant processes, causing a decrease in ROS [14] which, when added to the antioxidant properties of the molecule per se, causes decrease of OS, as observed in the present study. Likewise, additional to the antioxidant effect of HT, the increased concentration of HDL-c induced by the treatment helps to counteract OS. In this sense, it is known that HDL has antioxidant effects due to the presence of paraoxonase-1 and sphingosine-1-phosphate (S1P) [31], this increase in HDL-c will mitigate the OS status of the treated women.

It is important to note that OS reduction was observed in the placebo groups at three months, but this effect was not present at six months. It has been recognized that in clinical trials, a placebo effect can occur as a psycho-biological phenomenon attributable to the context in which the treatment is conducted [35]. Indeed, it has been reported a clinical trial using antioxidant vitamins vs. placebo in the elderly, which found that the placebo had a significant effect on OS due probably at motivation and an expectation of the treatment [36]. In the current work, the expectations of the participants included an improvement of their symptoms due to treatment, an improvement of their attitudes and the acquisition of a commitment to follow a therapy that positively influences OS; however, after three months the participants in the placebo groups did not notice any changes, thereby returning to high levels of OS.

Also, it is necessary to denote that, although the use of HT is still controversial, there is a statement about this topic on aspects of safety and disease prevention. This statement points out that the indication of HT is safe in women before age of 60 years or within 10 years after menopause [15], as were the ones we used in this study; therefore, we consider an additional benefit of this therapy on early menopause.

Finally, we note that this study has the limitation of a small sample size. However, its strength relies on being a double-blind clinical trial in which most of the potential biases were controlled, including that OS would be related to pro-oxidant lifestyle factors instead of the postmenopausal-MetS process, our main interest; and that the antioxidant action is solely attributable to HT and not to other antioxidants. This notion was discarded because the groups were similar, regarding these variables. However, an increased monitoring time and larger sample size are alternatives for confirming the results shown here. Another limitation is that of HDL functionality, especially cholesterol efflux capacity, which has been found more important than HDL-c concentration [37] but the techniques to evaluate this are not available in clinical practice [38]. Also, S1P is a lysophospholipid carried by HDL that exerts antioxidant properties, therefore improves HDL functionality [39] and has cardioprotective effect [40]; thus, HT may have reduced OS mediated by an increase in S1P levels, given that it raised the HDL-c concentration but the S1P levels were not measured.

## 4. Materials and Methods

### 4.1. Study Design

A randomized, double-blind, placebo-controlled trial was carried out on 100 postmenopausal women with intact uterus, in a community-dwelling from Mexico City, Mexico. The women were aged 45 to 59 years and had at least 12 months of spontaneous amenorrhea and/or serum estradiol levels less 25 pg/mL and follicle stimulating hormone (FSH) levels higher 50 mU/mL. They were invited to participate in the project “Menopause and oxidative stress” and were selected from 150 women who responded to the invitation. Eligibility criteria were healthy postmenopausal women or with MetS. MetS was defined according to criteria established in the Third Report of the National Cholesterol Education Program Expert Panel on Detection, Evaluation and Treatment of High Blood Cholesterol in Adults (Adult Treatment Panel III) [2]. They included: values greater than or equal to 1.65 mmol/L of TG; 6.05 mmol/L of fasting glucose; 88 cm of waist circumference and 130/85 mmHg of blood pressure; and also less than 1.3 mmol/L of high-density lipoprotein cholesterol (HDL-c). For the diagnosis of MetS the presence of three or more of these components was necessary.

The participants were not admitted into the study if any of the following criteria were present: cancer disease; history, or active presence of chronic renal; hepatic disease or thromboembolic disorders; cerebrovascular disease; history of depression; myocardial infarction or ischemic heart disease; as assessed by their medical history and physical examination. They were also excluded if they had taken an antioxidant supplement at least six months prior to the beginning of the study. None of the participants had previously received hormone therapy.

The women agreed to participate in the study after signing their informed consent. The Ethics Committee of the Universidad Nacional Autonoma de Mexico, Zaragoza Campus (Mexico City, Mexico) (FESZ/DEPI/097/13), approved the research protocol for this study.

### 4.2. Treatment Phase

The treatment allocation used the simple random method with a scientific calculator, the list of participants as previously discussed according to their study entry and separated by metabolic state: healthy or MetS. We formed four groups of 25 women each: group, HW receiving HT (1 mg/day of estradiol valerate plus 5 mg/10 day of medroxiprogesterone); group 2, HW taking placebo (pharmaceutical presentation similar to the treatment); group 3, MSW with HT; group 4, MSW with placebo. The treatment was taken by oral administration.

### 4.3. Follow-up Phase

Follow-up was for 6 months with a baseline measurement prior to initiation of therapy, at three and six months. Telephone contact was maintained with the participants every month, attending to collect endowments treatments also monthly. All participants had to return empty bottles in exchange for new ones with the following month′s treatment. A count of leftover tablets to verify compliance with treatment was performed. Elimination criteria were considered as treatment noncompliance in at least a month and a drop out of the study by any cause. All participants underwent a mammogram and cervical cytology (Pap) at the beginning and end of the study. Adverse events were recorded at each visit as the participant came to refill prescription.

### 4.4. Anthropometric Measurements and Blood Pressure

After clinical history and physical examinations were conducted, we performed the following anthropometric measurements: weight was measured while the woman was wearing underwear and a clinical gown and in a fasted state (after evacuation). A Torino^®^ scale (Tecno Lógica, Mexicana, Mexico, TLM^®^) was used and was calibrated before each weight measurement. Height was obtained with an aluminum cursor stadiometer graduated in millimeters. The woman stood barefoot, back and head in contact with the stadiometer in Frankfurt horizontal plane. Body mass index (BMI) was calculated by dividing weight (in kilograms) by squared height (in meters).

Blood pressure was measured in both arms 3 times in the morning, in a fasting condition, in a sitting position. A mercurial manometer was used to measure blood pressure and it was taken by medical technicians who had attended training sessions to standardize the procedures. The technicians were supervised in order to avoid possible biases in measurement.

### 4.5. Blood Sampling and Biochemical Assay

Blood samples were collected after a 12-h fasting period by venipuncture, placed in vacutainer/siliconized test tubes without additives and heparin used as anticoagulant agent (Becton-Dickinson, Mexico City, Mexico). We measured complete blood count in samples containing heparin in a Celly 70 autoanalyser (Chronolab, Mexico City, Mexico). Serum was obtained from samples without additives and was tested for glucose, uric acid, cholesterol, triglycerides, HDL-c, and albumin concentrations using a Cobas C111 analyzer (Roche Diagnostics, Basilea, Switzerland).

Glucose levels were measured with glucose oxidase method; uric acid levels by uricase colorimetric method; albumin levels by bromocresol green technique; cholesterol was analyzed using CHOD-PAP technique; TG by GPO-Trinder technique; whereas HDL-c were assessed employing CHOD-PAP technique after precipitation of low and very-low lipoproteins using a phosphotungstic acid/magnesium chloride solution. All reagents employed in biochemical tests were obtained from Roche Diagnostics. We included a high and normal control serum as the quality control (Roche Diagnostics). Low density lipoprotein cholesterol (LDL-c) was obtained with the standard formula of Friedewald. The intra- and inter-assay variation coefficients were less 5% in all determinations. Those tests were used to establish women health status and cut-off points for reference values for Mexican adults were determined at the Gerontologic Clinical Research Laboratory of the Universidad Nacional Autónoma de México (UNAM) Zaragoza Campus in Mexico City [41].

We measured estrogens and FSH levels to confirm postmenopausal state using a radioimmunoassay method (Siemens, Malvern, PA, USA) for estrogens, and chemiluminescence method (Siemens) for FSH. The within-run precision levels for these methods were 3.1% and 7.4%, respectively, and the estrogens analytical sensitivity was 8 pg/mL.

### 4.6. Oxidative Stress Measurement

Blood samples containing heparin we used to measure red blood cell SOD and GPx activities, plasma total antioxidant status (TAS), and plasma LPO concentration.

SOD activity was measured by the method that employs xanthine and xanthine oxidase (XOD) in order to generate superoxide radicals, which react with 2-(4-iodophenyl)-3-(4-nitrophenol)-5-phenyltetrazolium chloride (INT) to form a red formazan dye (Randox Laboratories, Ltd., Crumlin, County Antrim, UK). GPx was measured using the oxidation of glutathione (GSH) by cumene hydroperoxide in the presence of glutathione reductase and NADPH; oxidized glutathione (GSSG) is immediately converted into the reduced form with the subsequent oxidation of NADPH to NADP^+^ (Randox Laboratories, Ltd.). TAS quantification was done using 2,2-azino-bis (3-ethylbenzthiazoline-6-sulfonic acid, ABTS^+^) radical formation kinetics (Randox Laboratories Ltd.). The LPO concentration was measured with thiobarbituric acid reacting substances (TBARS) assay. It was performed as described by Jentzsch et al. [42] as we previously validated. In the TBARS assay, one molecule of malondialdehyde (MDA) reacts with two molecules of thiobarbituric acid (TBA) with production of a pink pigment with absorption at 535 nm. Amplification of peroxidation during the assay was prevented by the addition of the chain-breaking antioxidant butylated-hydroxytoluene (BHT). All the measures were performed in a Shimadzu UV-1601 UV-Vis spectrophotometer (Shimadzu corporation, Kyoto, Japan).

In addition, we calculated SOD/GPx ratio, and antioxidant gap with the equation [43]:

(1) GAP=TAS−[(albumin (µmol)×0.69)+uric acid (µmol)]

Alternative cut-off values of each parameter were defined on the basis of the 90th percentile of young healthy subjects: LPO ≥ 0.320 µmol/L; SOD ≤ 1.20 U/g·Hb; GPx ≤ 50.1 U/g·Hb; TAS ≤ 900 µmol/L, SOD/GPx ≥ 0.023; GAP ≤ 190 µmol/L. Uric acid cut-off value was median of reference interval (>268 µmol/L) for Mexican adult women [41]. An OS score (SS) was obtained ranging from 0 to 7, represented the severity of the biomarkers modifications; a score of 1 was given to each value higher or lower than the cut-off point established. A cut-off value of ≥4 was considered as OS.

Also we considered pro-oxidant factors across a structured questionnaire that included factors such as smoking, the consumption of caffeinated and alcoholic beverages and physical activity. We considered a pro-oxidant factor present when the following were noted: smoking ≥2 cigarettes/day, consumption of alcoholic beverages ≥2 glasses/day, consumption of caffeinated beverages >2 cups/day, and physical activity <30 min/day. All the women answered this questionnaire at baseline.

### 4.7. Statistical Analysis

Quantitative data were expressed as the mean ± standard error and they were compared using Student′s *t*-test between placebo and HT groups for each healthy woman and women with MetS. Qualitative data were analyzed using frequencies and percentages compared with *chi* *square* test; also a proportion of 95% confidence interval was obtained.

We calculated the percent change obtaining the difference between the percentage at baseline and after 3- or 6-month follow-up, this comparison was carried out with the McNemar *chi* *square* test.

To establish the effect of treatment, mean of SS was compared with repeated measure analysis of variance (ANOVA) test and paired *t*-test. A *p*-value < 0.05 was considered significant. The data were processed by use of the standard statistical software package SPSS V. 20.0 (IBM SPSS Statistics Armonk, NY, USA).

## 5. Conclusions

In conclusion, our results suggest that HT improves the lipid and oxidative alterations that occur in MetS in postmenopausal women, supporting the proposal that HT has an antioxidant effect, thus could be used as a complementary treatment option for improve MetS and OS.

## Figures and Tables

**Table 1:** Baseline parameters and oxidative stress markers of study groups.

Variable	Healthy		Metabolic Syndrome	
	Treatment (*n* = 23)	Placebo (*n* = 23)	Treatment (*n* = 23)	Placebo (*n* = 22)
Age (years)	52 ± 0.6	53 ± 0.7	52 ± 0.7	53 ± 0.9
Glucose (mmol/L)	5.0 ± 0.12	5.5 ± 0.47	6.8 ± 0.83	7.2 ± 0.66
Cholesterol (mmol/L)	5.5 ± 0.25	5.6 ± 0.33	5.9 ± 0.31	5.4 ± 0.23
Triglycerides (mmol/L)	1.79 ± 0.17	1.66 ± 0.14	2.42 ± 0.18	2.21 ± 0.14
HDL cholesterol (mmol/L)	1.64 ± 0.10	1.61 ± 0.07	1.33 ± 0.08	1.33 ± 0.06
LDL cholesterol (mmol/L)	3.0 ± 0.16	3.3 ± 0.33	3.5 ± 0.26	3.2 ± 0.18
Body mass index (kg/m^2^)	28.85 ± 0.96 *	25.93 ± 0.77	29.67 ± 0.97	30.85 ± 1.00
Waist circumference (cm)	93.0 ± 2.1	87.4 ± 2.6	98.1 ± 2.3	100.8 ± 2.4
Systolic blood pressure (mmHg)	117 ± 2.1	123 ± 3.2	128 ± 3.5	135 ± 4.4
Diastolic blood pressure (mmHg)	79 ± 2.1	81 ± 2.0	84 ± 1.7	86 ± 2.3
Oxidative stress marker				
Lipid peroxides (μmol/L)	0.341 ± 0.01	0.336 ± 0.01	0.356 ± 0.01	0.357 ± 0.01
Superoxide dismutase (U/g·Hb)	1.24 ± 0.02	1.20 ± 0.03	1.26 ± 0.05	1.21 ± 0.03
Glutathione peroxidase (U/g·Hb)	53.0 ± 2.7	50.8 ± 2.8	57.2 ± 4.2	52.9 ± 3.5
Total antioxidant status (µmol/L)	963 ± 41	1076 ± 51	1075 ± 42	1068 ± 43
SOD/GPx ratio	0.025 ± 0.002	0.025 ± 0.003	0.025 ± 0.003	0.025 ± 0.002
Antioxidant gap (μmol/L)	239 ± 41	334 ± 51	316 ± 52	298 ± 37
Uric acid (µmol/L)	259 ± 15	261 ± 17	279 ± 21	284 ± 19

* Student’s *t*-test, *p* < 0.05. SOD: superoxide dismutase; GPx: glutathione peroxidase. Data show mean ± standard error.

**Table 2:** Percentage of study women with metabolic alterations according to National Cholesterol Education Program Adult Treatment Panel III (NCEP-ATP III) criteria and pro-oxidant factors by study group.

Variable	Healthy		Metabolic Syndrome	
	Treatment (*n* = 23)	Placebo (*n* = 23)	Treatment (*n* = 23)	Placebo (*n* = 22)
Hyperglycemia (≥6.05 mmol/L)	2 (9%, 0%–21%)	3 (13%, 0%–26%)	8 (35%, 16%–54%)	13 (59%, 39%–79%)
Hypertriglyceridemia (≥1.65 mmol/L)	9 (41%, 21%–61%)	8 (35%, 16%–54%)	19 (83%, 68%–98%)	17 (77%, 60%–94%)
Hypoalphalipoproteinemia (<1.3 mmol/L)	4 (17%, 2%–32%)	3 (13%, 0%–26%)	13 (57%, 37%–77%)	8 (38%, 18%–58%)
Overweight (≥25 kg/m^2^)	15 (68%, 49%–87%)	13 (57%, 37%–77%)	20 (87%, 73%–100%)	20 (91%, 79%–100%)
Abdominal obesity (≥88 cm)	16 (70%, 51%–89%)	12 (52%, 32%–72%)	20 (87%, 73%–100%)	20 (91%, 79%–100%)
Hypertension (≥130/85 mmHg)	7 (30%, 11%–49%)	9 (39%, 29%–49%)	13 (57%, 37%–77%)	18 (82%, 66%–98%)
Current smokers (≥2 cigarettes/day)	2 (9%, 0%–21%)	1 (4%, 0%–12%)	3 (13%, 0%–26%)	4 (18%, 2%–34%)
Caffeinate beverages intake (≥2 cup/day)	5 (22%, 5%–39%)	4 (17%, 2%–32%)	8 (38%, 18%–58%)	6 (27%, 9%–45%)
Alcohol intake (>2 glasses/d)	1 (4%, 0%–12%)	0	2 (9%, 0%–21%)	1 (4%, 0%–12%)
Sedentary lifestyle *	12 (52%, 32%–72%)	13 (57%, 37%–77%)	15 (65%, 50%–80%)	15 (68%, 49%–87%)

* Sedentary if had <30 min/d of physical activity. Data show frequency (%, 95% confidence interval).

**Table 3:** Percentage change of metabolic syndrome markers after 6 months.

Metabolic Syndrome Marker	Healthy						Metabolic Syndrome					
	Hormone Therapy			Placebo			Hormone Therapy			Placebo		
	Basal (*n* = 5)	6 mo. (*n* = 23)	Diff.	Basal (*n* = 25)	6 mo. (*n* = 23)	Diff.	Basal (*n* = 25)	6 mo. (*n* = 23)	Diff.	Basal (*n* = 25)	6 mo. (*n* = 22)	Diff.
Hyperglycemia (≥6.05 mmol/L)	2 (9%)	2 (9%)	0	3 (13%)	6 (26%)	13%	8 (35%)	6 (26%)	−9%	13 (59%)	13 (59%)	0
Hypertriglyceridemia (≥1.65 mmol/L)	9 (41%)	10 (46%)	5%	8 (35%)	8 (35%)	0	19 (83%)	13 (57%)	–26% *	17 (77%)	20 (91%)	14%
Hypoalphalipoproteinemia (<1.3 mmol/L)	4 (17%)	4 (17%)	0	3 (13%)	8 (35%)	22%	13 (57%)	9 (39%)	−18%	8 (36%)	9 (41%)	5%
Abdominal obesity (≥88 cm)	16 (70%)	16 (70%)	0	6 (26%)	8 (35%)	9%	20 (87%)	20 (87%)	0	20 (91%)	20 (91%)	0
Hypertension (≥130/85 mmHg)	7 (30%)	3 (13%)	−17%	9 (39%)	9 (39%)	0	13 (57%)	12 (52%)	−5%	18 (82%)	18 (82%)	0

* McNemar *chi* *square* test, *p* < 0.05. Diff.: percent difference, 6 mo.: 6 months. Data show frequency (%).

**Table 4:** Oxidative stress markers after 6 months of follow-up.

Oxidative Stress Marker	Healthy				Metabolic Syndrome			
	Hormone Therapy		Placebo		Hormone Therapy		Placebo	
	Basal (*n* = 25)	6 mo. (*n* = 23)	Basal (*n* = 25)	6 mo. (*n* = 23)	Basal (*n* = 25)	6 mo. (*n* = 23)	Basal (*n* = 25)	6 mo. (*n* = 22)
Lipid peroxides (µmol/L)	0.340 ± 0.01	0.290 ± 0.01 ^a^	0.336 ± 0.01	0.348 ± 0.01	0.355 ± 0.01	0.282 ± 0.01 ^a^	0.357 ± 0.01	0.346 ± 0.01
SOD (U/g·Hb)	1.24 ± 0.03	1.26 ± 0.04	1.16 ± 0.02	1.19 ± 0.03	1.23 ± 0.05	1.26 ± 0.02	1.21 ± 0.03	1.22 ± 0.02
GPx (U/g·Hb)	53.0 ± 2.7	62.2 ± 2.7 ^b^	50.2 ± 3.0	52 ± 2.5	52.1 ± 4.8	62.9 ± 2.8 ^a^	52.5 ± 4.3	54.6 ± 3.2
TAS (µmol/L)	963 ± 41.5	1049 ± 40.4	1077 ± 50.7	1110 ± 48.2	1075 ± 41.8	1155 ± 48.3	1068 ± 43.2	1103 ± 60.0
Uric acid (µmol/L)	259 ± 15.2	240 ± 18.3	261 ± 17.3	275 ± 16.6	279 ± 21.0	253 ± 17.2	284 ± 19.4	300 ± 15.0
SOD/GPx ratio	0.023 ± 0.001	0.021 ± 0.001	0.025 ± 0.001	0.024 ± 0.001	0.026 ± 0.002	0.020 ± 0.001 ^a^	0.025 ± 0.002	0.023 ± 0.001
Antioxidant gap (µmol/L)	239 ± 40.9	276 ± 39.8	334 ± 51.0	357 ± 49.1	316 ± 52.0	460 ± 44.5 ^c^	298 ± 37.0	336 ± 56.1

Paired *t*-test: ^a^ *p* < 0.0001; ^b^ *p* = 0.001; ^c^ *p* < 0.05. SOD: superoxide dismutase; GPx: glutathione peroxidase; TAS: total antioxidant status; 6 mo.: 6 months. Data show mean ± standard error.
